# The Effect of Phenazine-1-Carboxylic Acid on Mycelial Growth of *Botrytis cinerea* Produced by *Pseudomonas aeruginosa* LV Strain

**DOI:** 10.3389/fmicb.2017.01102

**Published:** 2017-06-14

**Authors:** Ane S. Simionato, Miguel O. P. Navarro, Maria L. A. de Jesus, André R. Barazetti, Caroline S. da Silva, Glenda C. Simões, Maria I. Balbi-Peña, João C. P. de Mello, Luciano A. Panagio, Ricardo S. C. de Almeida, Galdino Andrade, Admilton G. de Oliveira

**Affiliations:** ^1^Laboratório de Ecologia Microbiana, Departamento de Microbiologia, Universidade Estadual de LondrinaLondrina, Brazil; ^2^Laboratório de Fitopatologia, Departamento de Agronomia, Universidade Estadual de LondrinaLondrina, Brazil; ^3^Laboratório de Produtos Fitoterápicos, Departamento de Farmácia e Farmacologia, Universidade Estadual de MaringáMaringá, Brazil; ^4^Laboratório de Micologia, Departamento de Microbiologia, Universidade Estadual de LondrinaLondrina, Brazil; ^5^Laboratório de Microscopia Eletrônica e Microanálise, Universidade Estadual de LondrinaLondrina, Brazil

**Keywords:** *Pseudomonas* secondary metabolites, gray mold disease, purification process, antifungal activity, bioactive compounds

## Abstract

One of the most important postharvest plant pathogens that affect strawberries, grapes and tomatoes is *Botrytis cinerea*, known as gray mold. The fungus remains in latent form until spore germination conditions are good, making infection control difficult, causing great losses in the whole production chain. This study aimed to purify and identify phenazine-1-carboxylic acid (PCA) produced by the *Pseudomonas aeruginosa* LV strain and to determine its antifungal activity against *B. cinerea*. The compounds produced were extracted with dichloromethane and passed through a chromatographic process. The purity level of PCA was determined by reversed-phase high-performance liquid chromatography semi-preparative. The structure of PCA was confirmed by nuclear magnetic resonance and electrospray ionization mass spectrometry. Antifungal activity was determined by the dry paper disk and minimum inhibitory concentration (MIC) methods and identified by scanning electron microscopy and confocal microscopy. The results showed that PCA inhibited mycelial growth, where MIC was 25 μg mL^-1^. Microscopic analysis revealed a reduction in exopolysaccharide (EPS) formation, showing distorted and damaged hyphae of *B. cinerea*. The results suggested that PCA has a high potential in the control of *B. cinerea* and inhibition of EPS (important virulence factor). This natural compound is a potential alternative to postharvest control of gray mold disease.

## Introduction

The search for natural products with a high potential in the field of sustainable agriculture (Hostettmann and Wolfender, 1997) is important. These compounds are broadly defined as active ingredients derived from plants, animals, or microorganisms, which reduce disease by stimulating plant defenses, direct antimicrobial activity, and/or decreasing biofilm formation (Romanazzi et al., 2012).

The potential of *Pseudomonas* species to suppress plant pathogens is well known (Dowling and O’Gara, 1994; Raaijmakers et al., 1997; Haas and Défago, 2005), and secondary metabolites produced by *Pseudomonas* spp. show strong bioactivity, including phenazines, pyrrolnitrin-type antibiotics, pyo compounds, indole derivatives, peptides, glycolipids, lipids, and aliphatic compounds (Fuller et al., 1971; Leisinger and Margraff, 1979; Ligon et al., 2000; Raaijmakers et al., 2002, 2006; Haas and Keel, 2003; Paulsen et al., 2005; Gross and Loper, 2009).

Phenazine is a heterocyclic nitrogen-containing secondary metabolites produced by pseudomonads (Price-Whelan et al., 2006) and has great potential for use as antifungal against many fungal species, such as *Gaeumannomyces graminis* var. *tritici, Fusarium oxysporum, Pythium* spp., *Rhizoctonia solani, Gibberella avenacea, Alternaria* spp., and *Drechslera graminea* (Mavrodi et al., 2006).

The fungus *Botrytis cinerea* is a plant necrotrophic pathogen that colonizes senescent or dead plant tissues and causes gray mold in plant and softening in fruits. The hyphae infect plant tissues through wounds or natural openings and spread from dead to healthy tissues (El Oirdi and Bouarab, 2007). *B. cinerea* infects different plant tissues of various crops, including tomato (*Solanum lycopersicum*), potato (*Solanum tuberosum*), grapes (*Vitis vinifera*), and strawberry (*Fragaria* spp.), causing great economic losses, at pre- or postharvest (Coley-Smith et al., 1980).

Gray mold management is based on chemical control with fungicides that may cause contamination in the environmental as well as in produce in the postharvest period, cause misshapen fruits due to time of application and resistance of the pathogen to common fungicides (Kovah et al., 2000; Myresiotis et al., 2007).

In modern agriculture, the use of synthetic fungicides is frequent and essential to ensure a good crop yield (Shephard, 1987; Knight et al., 1997). On the other hand, microbial metabolites may help to overcome pesticide resistance and contamination problems due to the versatility in structure and low toxicity to non-target organisms (Tanaka and Omura, 1993). The bioactive compound phenazine-1-carboxylic acid (PCA) produced by the *P. aeruginosa* LV strain is first described here as an antifungal against *B. cinerea*, with ultrastructural changes, and could help in the control of gray mold.

## Materials and Methods

### Chemicals and Culture Media

All chemical products used for extraction and purification were of analytical grade. The silica gel thin layer chromatography (TLC) sheets were from Macherey-Nagel GmbH & Co. KG), and culture media from Becton Dickinson and Co. Chemicals used for antimicrobial assays were purchased from Sigma–Aldrich.

### Bacterial Culture and Plant Pathogens

*Pseudomonas aeruginosa* strain LV was isolated from old citrus canker lesion on the leaves of orange plants (*Citrus sinensis* cv. Valence) in Astorga, Brazil (Rampazo, 2004), deposited in GenBank under accession number GQ342301 (Kerbauy et al., 2016). The strain was cryopreserved in liquid nitrogen in sterilized distilled water (SDW) plus 20% glycerol. The production of metabolites was patented (Andrade, 2008), and *P. aeruginosa* LV strain was grown on medium containing 0.5% peptone, 0.3% meat extract, 0.01% CuCl_2_⋅2H_2_O; pH 6.8 at 28°C for 10 days in sterilized atmospheric-pressure air.

*Botrytis cinerea* was isolate from strawberry fruits with typical symptoms and identified through its reproductive structures under the microscope and was deposited in the Microbial Culture Collection of the Plant Pathology Laboratory, Department of Agronomy, State University of Londrina. *B. cinerea* was cultivated on PDA in Petri dishes at 28°C for 7 days. The fungal inoculum was prepared by washing spores from mycelia, and the spores were removed and resuspended in 2 mL of SDW. The spore suspension was filtered with sterile gauze to remove the mycelia and was adjusted to 5 × 10^6^ conidia mL^-1^ for further assays.

### Extraction and Purification of Antifungal Compound

The LV strain was cultured in bottles (Nalgene, Rochester, NY, United States) with 15 L of culture broth prepared as described above. After 10 days, the culture was centrifuged at 9,000 rpm for 15 min 4°C to obtain a cell-free supernatant. The metabolites were extracted five times from the supernatant using two volumes of dichloromethane each time (100 mL of supernatant and 200 mL of dichloromethane) and was designated the dichloromethane phase (DP). DP was concentrated in a rotary evaporator (Büchi 215R, Switzerland) to obtain 900 mg DP.

Dichloromethane phase was purified by flash chromatography (FC), and a sample was dissolved in 2 mL of dichloromethane and mixed with silica gel before being packed in a chromatography column (0.04–0.063 mm, Merck) to prepare the metabolite-silica gel slurry. Before chromatography, the column was air-dried by complete evaporation of the solvent at room temperature. The column (35 cm length and 1 cm diameter) was coupled to a low-pressure pump and washed using a mobile phase (v/v) with different proportions of dichloromethane:ethyl acetate (100:0, 95:05, 50:50, and 0:100 v/v). Approximately 1 mL of the eluate was collected in tubes and monitored by TLC. Similar fractions were mixed according to TLC analysis, and seven combined fractions were obtained (FC1 to FC7). The fractions were concentrated using a rotary evaporator (Büchi 215R) and tested for antifungal activity against *B. cinerea* with dry paper disk method.

The FC3 fraction was again purified by FC as described above, except the mobile phase was dichloromethane:ethyl ether (100:0, 95:05, 50:50, and 0:100; v/v). Fractions of approximately 1 mL of the mobile phase were collected in tubes and monitored by TLC. Similar fractions were combined, where 11 combined fractions were obtained and antifungal activity was determined by the paper disk method. The fraction with the highest antifungal activity against *B. cinerea* was purified and confirmed by preparative high performance liquid chromatographic (prep HPLC).

### HPLC Analysis

The peak was separately collected from the prep HPLC system with a C18 reversed-phase column (Prep C18, 10 mm × 250 mm, Agilent^®^, United States). The mobile phase was water:acetonitrile (50:50; 20:80; 0:100 v/v) at a flow rate of 2 mL min^-1^ and each peak was detected between 250 and 210 nm. The collected peak was assayed to determine antifungal activity against *B. cinerea*. Crystals were developed with one milligram of pure compound dissolved in chloroform in a vial at room temperature, which was left to slowly evaporate.

### TLC Analysis

The fractions obtained were carried out on silica gel 60 F254 plates, and each chromatograms was spotted on Petri dishes after to carefully dried for complete removal of the solvents. The chromatograms were developed in dichloromethane:ethyl acetate:methanol (45:45:10) and/or ethyl acetate:dichloromethane (95:5). The spots were viewed under ultraviolet light at 254 and 366 nm.

### Chemical Analysis

The pure compounds were dissolved in CDCl_3_ at 1,000 μg mL^-1^ and the mass spectrum was recorded using an ESI-MS Quattro LCZ (Micromass Manchester, United Kingdom). ^1^H and ^13^C nuclear magnetic resonance spectra were recorded in solution using a Bruker, Avance III 400 MHz instruments. The UV/Vis absorption spectrum was measured from 220 to 400 nm with a Thermo Electron Corporation BIOMATE 3 spectrophotometer.

### Biological Assays

#### Agar Diffusion

The disks were impregnated with 250 μg disk^-1^ of semi-purified fractions and 100 μg disk^-1^ purified compound with three replicates. The fungi were added as 6 mm-diameter mycelial plugs on 20 mL of PDA, and fungal growth was recorded after 4 days of incubated at 20°C (full fungal growth in the control plates). The chemical solvent dichloromethane was considered the negative control. The experiment was repeated three times and the antifungal effect was determined by measuring (mm) the inhibition halos formed around the disk.

#### Minimum Inhibitory Concentration (MIC)

The MIC values for *B. cinerea* were determined using a 10-fold serial dilution method in Petri dishes with PDA, where 10 mL of PDA were mixed with respective concentrations of purified compound in the melted agar as follows (0, 0.7, 1.5, 3.1, 6.2, 12.5, 25, and 50 μg mL^-1^). Disks of 6 mm diameter with *B. cinerea* mycelium were placed on culture medium surface with respective concentrations of pure compound, and mycelial growth was evaluated after 4 days incubation at 20°C. Each treatment was performed in triplicate, and the percentage of mycelial growth inhibition (MGI) was calculated the according to Yahyazadeh et al. (2008):

$$\begin{array}{c} MGI(\%)=\left[\frac{d_{c}-d_{t}}{d_{c}}\right]\times 100 \end{array}$$

where *d*_c_ (mm) is mean colony diameter in the control and *d*_t_ (mm) is mean colony diameter of each treatment. The 50 and 80% effective dose (ED50 and ED80) was determined by regression analysis when the growth was reduced by 50 or 80% and compared with control, respectively. The lowest concentration that completely inhibited the growth of fungus was considered the minimum inhibitory concentration (MIC).

### Microscopic Analysis

#### Scanning Electron Microscopy (SEM)

Fungal cultures grown for 4 days in PDA and treated with different concentrations of PCA (0, ED50 and ED80) were used for all SEM observations. Plugs 6 mm in diameter were cut from cultures and placed in vials containing 3% glutaraldehyde and 2% paraformaldehyde in 0.1 M sodium cacodylate buffer (pH 7.2) at 4°C. Samples were kept in this solution for 4 h for fixation and were then washed with 0.1 M sodium cacodylate buffer (pH 7.2) for 10 min three times. Subsequently, the samples were dehydrated in an ethanol series (70, 80, 90, and 100%) for 10 min three times. Samples were critical-point dried with CO_2_ (BALTEC CPD 030 Critical Point Dryer), coated with gold (BALTEC SDC 050 Sputter Coater) and observed in a FEI Quanta 200 SEM operating at 30 kV.

#### Confocal Microscopy

Four-day-old fungal cultures grown on PDA treated as described above were used for all confocal microscopy observations. Mycelial plugs 6 mm in diameter were cut from cultures grown on PDA plates and promptly placed in wells of a 24-wells plate containing fixative (3.7% formaldehyde [Vetec] in phosphate buffered saline [PBS], pH 7.2) and incubated at room temperature for 2 h. Next, the samples were washed twice with PBS and stained as follows. The mycelial samples were incubated in 10 mg mL^-1^ Calcofluor White (Sigma-Aldrich) and 0.1 M Tris-hydrochloride (pH 9.0 [Roth]) for 20-min at room temperature and washed three times with ultra-pure water. Afterward, the exopolysaccharide matrix (EPS) was stained with 12.5 mg mL^-1^ fluorescein-conjugated concanavalin A (Molecular Probes) in PBS for 45 min at room temperature. After washing three times with PBS, the mycelial plugs were inverted on a slide with 6 μL of glycerol (20% in PBS) and covered with a coverslip. Confocal analysis was performed under an SP8 Leica inverted microscope. All samples were exposed to the same light intensity and for the same time. A representative photograph of each condition was selected.

## Results

### Isolation and Purification of the Antifungal Compound

**FIGURE 1:**
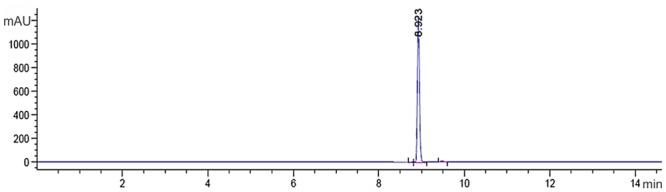
Chromatogram compound PCA C18 RP-HPLC column with detection at 250 nm. The presence of a single peak at 8.92 min indicates that the high purity compound. Absorbance × Retention Time.

Using 30 L of cell-free supernatant, 1,800 mg of crude extract was obtained by extraction with dichloromethane. The crude extract was fractionated by silica gel column chromatography with dichloromethane:ethyl acetate as mobile phase. The Fraction FC3 was eluted again with dichloromethane:ethyl acetate [50:50], and the antifungal compound was extracted (140 mg). During the process, fungal activity was monitored with agar diffusion assays. In analytical TLC, the antifungal compound was determined in a spot with an *Rf* of 0.62. The FC3 fraction was separated by FC using dichloromethane:ethyl ether, where the antifungal compound (60 mg) was eluted at around 95:5 eluent and also monitored by agar diffusion assays. In analytical TLC, the antifungal compound was determined in a band with an *Rf* of 0.62. Prep HPLC yielded a single symmetrical peak at 250 nm with a retention time of 8.92 min (**Figure 1**), confirming the purity of the compound. The yield from 1,800 mg of crude extract was 90 mg of the antifungal compound, which was a greenish-yellow, needle-shaped, crystalline solid, observed after complete drying. The crystals were completely soluble in DMSO, CH_3_OH, and CHCl_3_ and insoluble in water.

### Identification of the Pure Antifungal Compound

**FIGURE 2:**
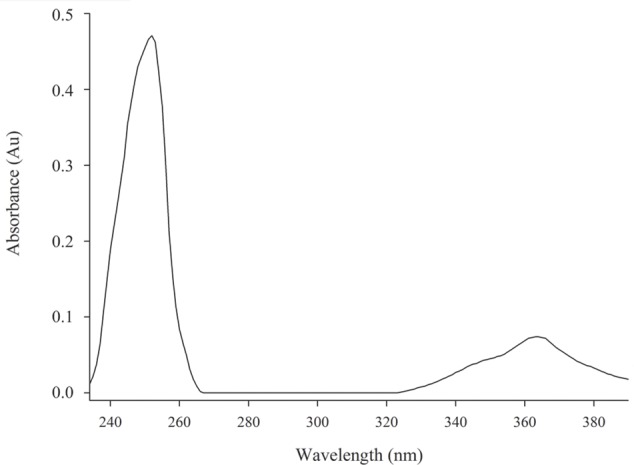
Spectrum UV/Vis from PCA sample. Strong absorption peak at 252 nm accompanied by a broad peak at 365 nm with a shoulder at 354 nm. Absorbance × Wavelength.

**FIGURE 3:**
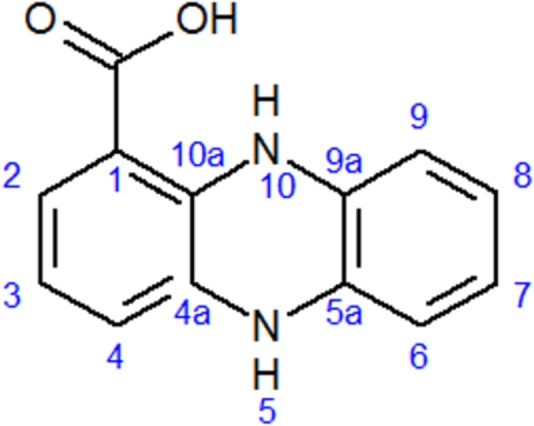
Structure of phenazine-1-carboxylic acid (PCA).

The chemical structure of pure antifungal compound was determined by NMR analysis (Supplementary Figures S1, S2 and **Table 1**) and ESI-MS data (Supplementary Figure S3). The mass spectrum displayed a molecular ion peak at *m/z* 247.0513 [M+Na]^+^ (Supplementary Figure S3). The presence of a phenazine moiety in the structure was further supported by a strong absorption peak at 252 nm followed by a broad peak at 365 nm with a shoulder at 354 nm in UV/Vis spectral analysis (**Figure 2**). The ^1^H and ^13^C NMR spectral data of pure antifungal compound and their assignments are shown in **Table 1**. On the basis of all spectral data and that proposed by Lee et al. (2003), the structure of the pure antifungal compound was determined to be PCA with molecular formula of C_13_H_8_N_2_O_2_ (**Figure 3**).

**Table 1:** ^13^C and ^1^H spectral data of phenazine-1-carboxylic acid in CDCl_3_.

N°	^13^C, δ_c_^a^	^1^H, δ_H_^b^ (*m^c^, J* in Hz)
1	125.08	–
2	130.12	8.56 (dd, 8,8; 2.49)
3	137.62	8.31 – 8.40 (m)
4	135.13	9.01 (dd, 7.0; 2.41)
4a	140.1	–
5a	142.89	–
6	131.74	7.99 – 8.09 (m)
7	128.01	8.31 – 8.40 (m)
8	130.3	8.31 – 8.40 (m)
9	133.22	7.99 – 8.09 (m)
9a	143.6	–
10a	144.14	–
COOH	165.93	15.62 (s)

*^a^100.61 MHz, chemical shift in ppm ^b^400 MHz, chemical shift in ppm ^c^Abbreviations of signal multiplicity are: (s), singlet; (dd), doublet of doublets; (m), multiplet.*

### Antifungal Activity of PCA against *B. cinerea*

During of all purification steps the fractions obtained were monitored by agar diffusion assays including the pure compound. The fractions FC3 and PCA showed significant antimicrobial activity while the other fractions did not show any antifungal activity. During the purification process, the antifungal activity against *B. cinerea* increased, and little difference was observed between FC3 (12 mm) and PCA (15 mm) in the disk diffusion assays.

**FIGURE 4:**
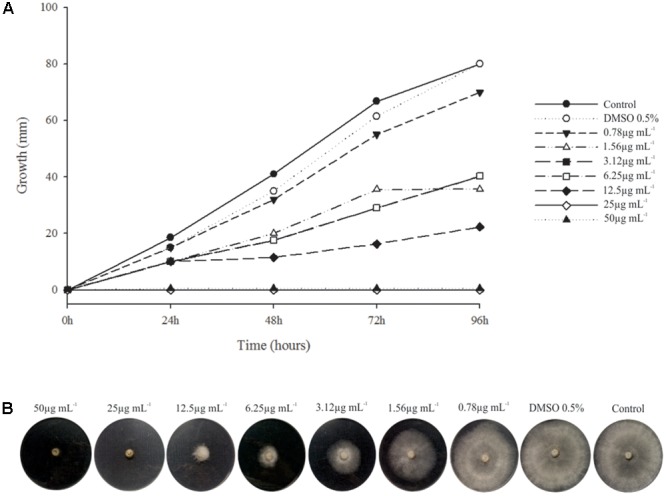
Minimum Inhibitory Concentration (MIC) PCA × *B. cinerea*. **(A)** Graphic of the relation of fungus growth × incubation time with different concentration of PCA. Fungus growth decreased from the concentration of 0.78 μg mL^-1^ of PCA and inhibition complete of mycelial growth from 25 μg mL^-1^. **(B)** MIC assay after 96 h of incubation at 20°C.

Phenazine-1-carboxylic acid at MIC (25 μg mL^-1^) clearly inhibited mycelial growth of *B. cinerea*, and the 50 and 80% effective doses (ED50 and ED80) were 3.12 and 12.5 μg mL^-1^, respectively (**Figure 4**).

### Scanning Electron Microscopy (SEM)

**FIGURE 5:**
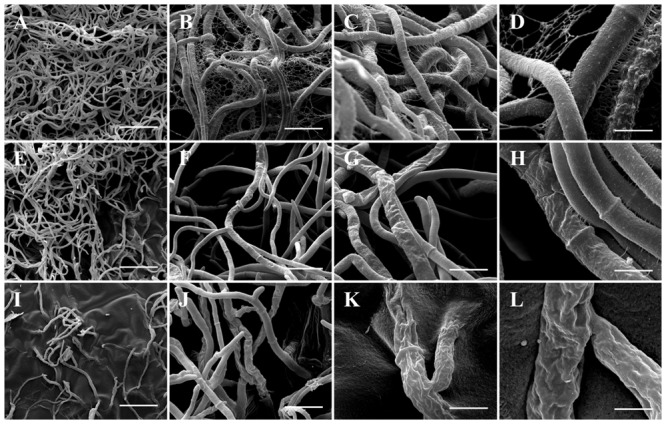
Scanning electron microscopy images of antifungal effect of PCA against *B. cinerea*. **(A–D)** Control (not treated with PCA) 96 h after inoculation; large number of hyphae and demonstrating hyphae with typical structure, smooth surface, and presence of EPS. **(E–H)** Treatment with 3.12 μg mL^-1^ PCA [ED50] 96 h after inoculation; inhibition the growth of *B. cinerea* by deforming the structure of fungal hyphae and decrease of the EPS formation. **(I–L)** Treatment with 12.5 μg mL^-1^ PCA [ED80] 96 h after inoculation; hyphae network was decreased, morphological alternations were very evident and was not possible to observe the presence of EPS. In the magnitudes of 400× (**A,E,I**: bar 75 μm), 3,000× (**B,F,J**: bar 20 μm), 6,000× (**C,G,K**: bar 10 μm) and 12,000× (**D, H, L**: bar 5 μm).

The effect of PCA on *B. cinerea* growth was strong, and the mycelia obtained from the edge of a *B. cinerea* colony in the control (non-treated) showed hyphae with typical “net” structure, smooth surface and presence of EPS (**Figures 5A–D**). In the presence of 3.12 μg mL^-1^ PCA, the hyphae lost smoothness and formed unusual surface bulges, indicating that PCA inhibited *B. cinerea* growth by causing deformation of hyphal structure and decreases in EPS production (**Figures 5E–H**). PCA at 12.5 μg mL^-1^ caused a great decrease in the amount of hyphal network and morphological changes, along with the absence of EPS (**Figures 5I–L**). The results showed that PCA distorted and damaged *B. cinerea* hyphae and that fungal growth was inhibited.

### Confocal Microscopy

**FIGURE 6:**
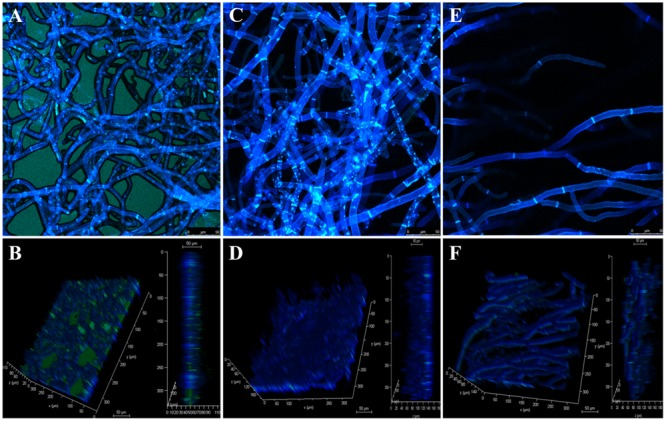
Confocal laser scanning micrographs of antifungal effect of PCA against *B. cinerea*. The fungal mycelia was stained in blue with Calcofluor White and the EPS was stained in green with Concanavalin A–fluorescein conjugated. **(A,B)** Control untreated with PCA. **(C,D)** Treatment with 3.12 μg mL^-1^ PCA [ED50]. **(E,F)** Treatment with 12.5 μg mL^-1^ PCA [ED80]. **(B,D,F)** Vertical section and reconstructed 3-D image of the fungal mycelia.

Confocal microscopy showed that EPS production decreased when fungi were treated with PCA (**Figure 5**), when compared with control in which an extensive mycelial growth was observed (**Figures 6A,B**). The addition of 3.12 μg mL^-1^ PCA (ED50) inhibited mycelial EPS production but did not affect mycelial density (**Figures 6C,D**). PCA at ED80 decreased EPS production and mycelial density (**Figures 6E,F**).

## Discussion

In natural environments, many microorganisms coexist in close proximity, leading to many types of interactions. For their survival, microorganisms develop an offensive strategy (production of metabolites with antimicrobial activity) to compete which others. Many classes of antimicrobial compounds produced as secondary metabolites by microorganisms have been reported (Bérdy, 2005; Mousa and Raizada, 2013), including polyketides, non-ribosomal peptides, terpenoids, heterocyclic nitrogenous compounds, volatile compounds, bacteriocins, and lytic enzymes, as well.

Secondary metabolites produced by microorganisms are alternative antimicrobial agents. The potential of pseudomonads to suppress plant pathogens is well known (Dowling and O’Gara, 1994; Raaijmakers et al., 1997; Haas and Défago, 2005), usually by the production of secondary metabolites that show strong antibiotic activity, including phenazines, pyrrolnitrin-type antibiotics, pyo compounds, indole derivatives, peptides, glycolipids, lipids, and aliphatic compounds (Fuller et al., 1971; Leisinger and Margraff, 1979; Ligon et al., 2000; Raaijmakers et al., 2002, 2006; Haas and Keel, 2003; Paulsen et al., 2005; Gross and Loper, 2009; de Oliveira et al., 2016).

The present study determined the structure based on spectral data, and antifungal activity of PCA against *B. cinerea in vitro* assay, showing morphological changes in hyphal as well as reduction of the EPS production. Others authors using a fraction which contain PCA show antibiotic activity against many species of Gram-negative bacteria (Lopes et al., 2012; Góis et al., 2013; Vasconcellos et al., 2014; Spago et al., 2014; Murate et al., 2015; de Oliveira et al., 2016) which is caused by metallo antibiotic presence but not for PCA. When the PCA was tested no effect was observed against bacteria. However, PCA showed fungicide effect which is observed in others studies (Puopolo et al., 2013; Huang et al., 2016).

Phenazine-1-carboxylic acid at 3.12 μg mL^-1^ reduced the mycelial growth of *B. cinerea* by 50%, where it was 25 times more effective than phenazine-1-carboxamide (PCN) (Zhang et al., 2015). This study screened PCA, a new agent that has not been previously used in pure form in fruit production. To a certain extent, this study alleviated the problem regarding *B. cinerea* drug resistance. Meanwhile, the results indicated that PCA has the potential to aid in the development of new control agents against gray mold. However, only *in vitro* experiments were performed in this study, and these types of experiments differ from field experiments.

Scanning electron microscopy showed deformation of the fungal hyphae after exposure to the bioactive compound in agar, where hyphae appeared shriveled up. This characteristic has been demonstrated in *B. cinerea* treated with eugenol (100 mg L^-1^), which according to studies may be related to the production of reactive oxygen species (ROS), affecting membrane permeability (Wang et al., 2010). As a pyocyanin, PCA has the capacity to reduce NADH and glutathione oxidation, which can occur with increased levels of cellular oxidation and destabilization of the mitochondria. With mitochondrial destabilization there is a decrease in the coupling efficiency on the electron transport chain, thus forming ROS intermediates, which can lead to cellular oxidative stress and alteration of membrane permeability (Wang et al., 2010; Briard et al., 2015), but membrane permeability and oxidative stress tests have to be conducted to prove this hypothesis.

In SEM and confocal microscopy, *B. cinerea* treated with the ED50 of PCA reduced EPS production significantly, and at ED80 there was no evidence of any EPS, which was confirmed by confocal microscopy. According to El Oirdi et al. (2011), EPS produced by *B. cinerea* activates the salicylic acid pathway, which in turn antagonizes the jasmonic acid signaling pathway, allowing the fungus to develop the disease in tomato. This event is a strategy used by *B. cinerea* to overcome the plant defense system and spread within the host. The action of PCA in reducing EPS produced in *B. cinerea*, demonstrated in this study by electron and confocal microscopy, will probably decrease the pathogenicity and necrotic activity of the pathogen in fruits. Accordingly, PCA can act on two fronts against *B. cinerea*: first, as a direct antifungal agent and second as an inhibitor of EPS, which is directly related to the success of infection in the fruit. However, further experiments with plants and fruits are needed, but this was not the objective of the present study.

## Conclusion

In conclusion, PCA has the advantages of high efficiency, low production costs and two forms of bioactivity against *B. cinerea*, as a direct antifungal agent and EPS inhibitor. This natural compound is a potential alternative for controlling gray mold disease.

## Author Contributions

AS, GA, and AdO designed the study protocol, and participated in its design and coordination. AS, MN, MdJ, AB, CdS, and GS, carried out the antimicrobial and purification assays. AS, RdA, and AO carried out the microscopy assays. AS, MB-P, JdM, LP, RdA, GA, and AdO contributed to drafting the manuscript and/or critically revising the paper and intellectual content. All authors read and approved the final manuscript.

## Conflict of Interest Statement

The authors declare that the research was conducted in the absence of any commercial or financial relationships that could be construed as a potential conflict of interest.
