# Supplementary material for: The Effect of Phenazine-1-Carboxylic Acid on Mycelial Growth of Botrytis cinerea Produced by Pseudomonas aeruginosa LV Strain
Source: Front Microbiol. 2017 Jun 14;8:1102. doi: 10.3389/fmicb.2017.01102 (PMC5469906; doi:10.3389/fmicb.2017.01102)

Supplementary Material

**Inhibition of *Botrytis cinerea* mycelial growth and exopolysaccharide production by the natural product phenazine-1-carboxylic acid**

**Ane Stefano Simionato^1^, Miguel Octavio Pérez Navarro^1^, Maria Luiza Abreu de Jesus^1^, André Riedi Barazetti^1^, Caroline Santos da Silva^1^, Glenda Cavalari Simões^1^, Maria Isabel Balbi Peña^2^, João Carlos Palazzo de Mello^3^, Luciano Aparecido Panagio^4^, Ricardo Sérgio Couto de Almeida^4^, Galdino Andrade^1^, Admilton Gonçalves de Oliveira ^1*^**

^1^Laboratório de Ecologia Microbiana, Departamento de Microbiologia, Universidade Estadual de Londrina, Londrina, Paraná, Brazil.

^2^Laboratório de Produtos Fitoterápicos, Departamento de Farmácia e Farmacologia, Universidade Estadual de Maringá, Maringá, Paraná, Brazil.

^3^Laboratório de Micologia e Métodos Alternativos ao Uso de Animais, Departamento de Microbiologia, Universidade Estadual de Londrina, Londrina, Paraná, Brazil.

*Corresponding author: Admilton Gonçalves de Oliveira, Universidade Estadual de Londrina, Centro de Ciências Biológicas, Departamento de Microbiologia. CEP 86051-990, Londrina, Brazil.

E-mail: admilton@uel.br

**Keywords: *Pseudomonas* secondary metabolites, gray mold disease, purification process, antifungal activity, bioactive compounds.**

**Supplementary Figures**

**Fig. S1** ^1^H spectral data of phenazine-1-carboxylic acid in CDCl_3_

**Fig. S2** ^13^C spectral data of phenazine-1-carboxylic acid in CDCl_3_

**Fig. S3** Mass spectral data of phenazine-1-carboxylic acid


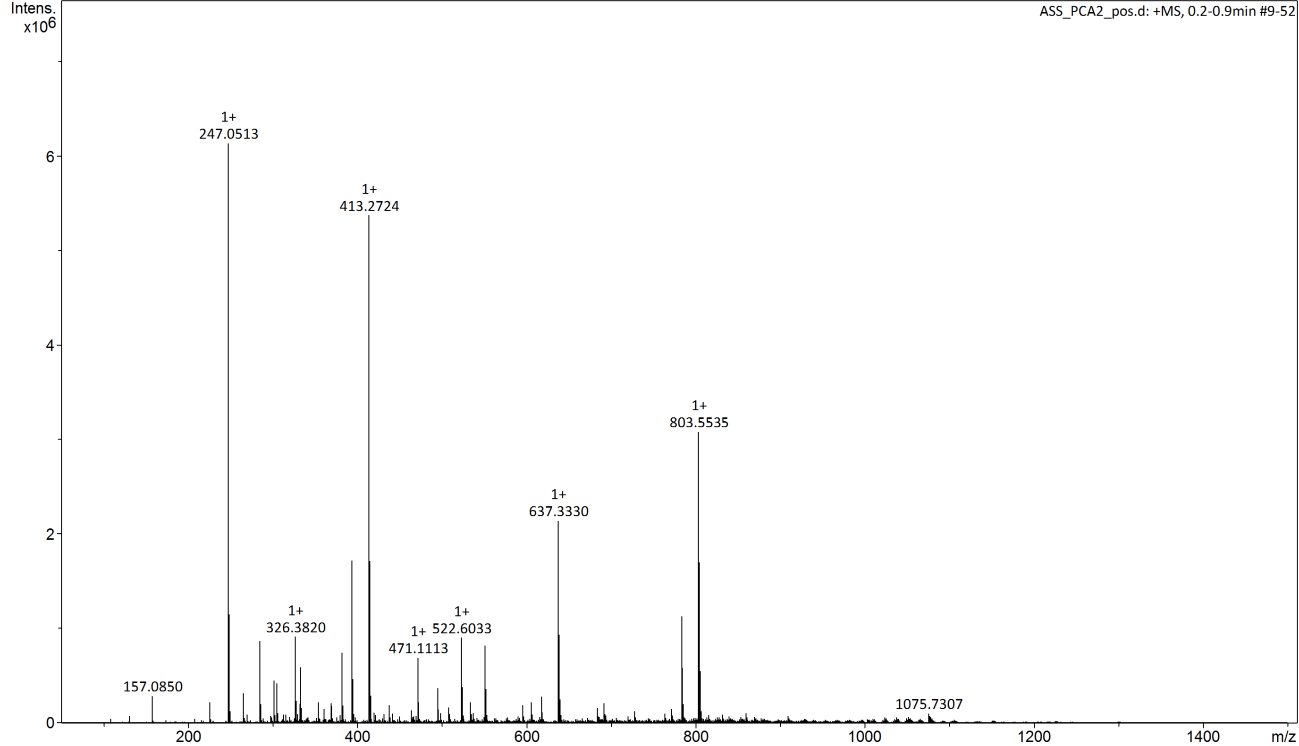

Supplement: Supplementary file 1 [file Data_Sheet_1.DOCX]
